
JOURNAL INFORMATION
==============================
NLM Title Abbreviation: ScientificWorldJournal
Journal ID: ScientificWorldJournal
NLM Journal ID: 101131163
Journal ID: TSWJ

The Scientific World Journal

ISSN: 2356-6140
EISSN: 1537-744X
Publisher: Wiley

ARTICLE INFORMATION
==============================
PMCID: PMC5763677
PMID: 21170484
DOI: 10.1100/tsw.2010.206
Article ID: 186458
Article version: 1
Subjects: Clinical Images

Lepromatous Leprosy: A Commonly Misdiagnosed Disease

Yaghoobi Reza 1
Feily Amir 1 *
Ranjbari Nastaran 2
Lotfi Jalal 1
Yaghoobi Elena 2
1 Department of Dermatology, Jundishapur University of Medical Sciences, Ahvaz, Iran
2 Department of Pathology, Jundishapur University of Medical Sciences, Ahvaz, Iran
*Amir Feily: dr.feily@yahoo.comAcademic Editor: Daniel Batlle

Publication date: 2010
Electronic publication date: 2010 Dec 14
Volume: 10
First page: 2348
Last page: 2349
Received 2010 Sep 4; Revised 2010 Oct 4; Accepted 2010 Oct 6
Copyright: Copyright © 2010 Reza Yaghoobi et al.
Copyright year: 2010
License: This is an open access article distributed under the Creative Commons Attribution License, which permits unrestricted use, distribution, and reproduction in any medium, provided the original work is properly cited.
License URL: https://creativecommons.org/licenses/by/3.0/

Keywords: lepromatous leprosy, endemic disease

==============================
Clinical Image
*TheScientificWorldJOURNAL* (2010) 10, 2348–2349
ISSN 1537-744X; DOI 10.1100/tsw.2010.206

TheScientificWorldJOURNAL
www.thescientificworld.com

### Lepromatous Leprosy: A Commonly Misdiagnosed Disease

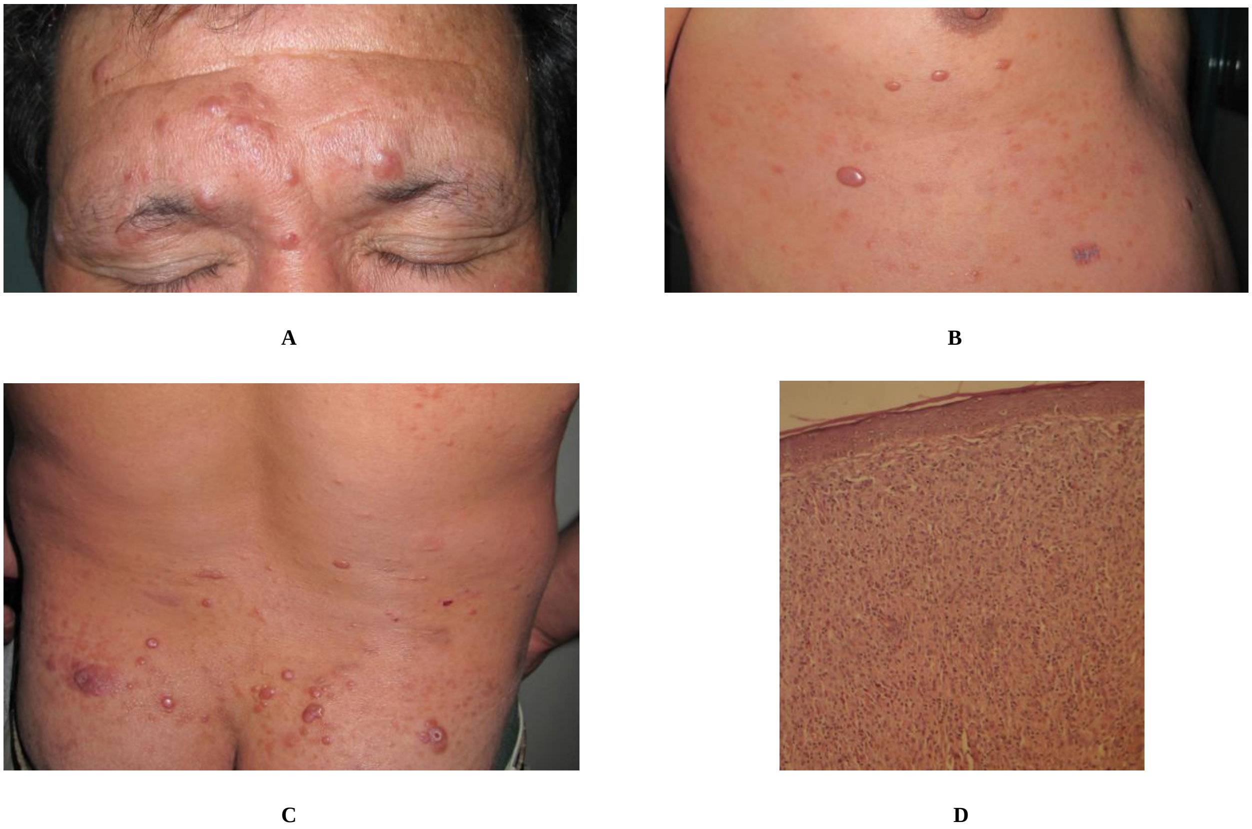

**FIGURE 1.** Erythematous/coppery papules and nodules appearing on the face and body (A–C), with no apparent granuloma (D).

Reza Yaghoobi[1], Amir Feily[1,*], Nastaran Ranjbari[2], Jalal Lotfi[1], and Elena Yaghoobi

*[1]Department of Dermatology and [2]Department of Pathology, Jundishapur University of Medical Sciences, Ahvaz, Iran*

**E-mail**: Yaghoobi_rz@yahoo.com; dr.feily@yahoo.com; saminjan@yahoo.com; sjlotfi@yahoo.com

*Received September 4, 2010; Revised October 4, 2010; Accepted October 6, 2010; Published December 14, 2010*

**KEYWORDS**: lepromatous leprosy, endemic disease

*Corresponding author.
©2010 with author.
Published by TheScientificWorld; www.thescientificworld.com

A 42-year-old man was referred to our department for treatment with an 8-year history of cutaneous nodules of the face, back, trunk, and buttocks. Physical examination was notable for multiple painless erythematous/coppery papules and nodules with loss of the lateral eyebrows (Fig. 1A–C). There was no variation in the cutaneous sensations of temperature, touch, and pain. The patient came from Afghanistan, an area in which lepromatous leprosy is endemic. Histopathologic examination showed infiltration of foamy histocytes and macrophages extending to the subcutaneous fat separated from the epidermis by the narrow granz zone. Apparently there was no granuloma formation (Fig. 1D). In a mucosal smear from his nose and acid fast staining, numerous acid-fast bacilli were seen and a diagnosis of lepromatous leprosy was made. Cutaneous clinical lesions of lepromatous leprosy include cutaneous/subcutaneous firm, translucent, erythematous/coppery, and shiny papules and nodules appearing over an apparently normal skin[1]. Ocular complications are found in 73% of the patients and loss of eyebrows is the most frequent occurrence[2]. A number of skin diseases may be confused with leprosy. The differential diagnosis of the infiltrated plaque or nodules includes leishmaniasis, syphilis, sarcoidosis, lymphoma, and cutaneous tuberculosis[3]. If any physician sees a patient from an area where this infection is endemic (Asia, sub-Saharan Africa, South and Central America, the Pacific Islands, and the Philippines)[4] and who has unexplained skin lesions, it is important to consider leprosy. This patient was treated successfully with World Health Organization (WHO)–recommended multibacillary multidrug therapy (MBMDT). The skin lesions resolved with minimal scarring.

### REFERENCES

1. Sehgal, V.N., Aggarwal, A., Srivastava, G., Sharma, N., and Sharma, S. (2005) Evolution of histoid leprosy (de novo) in lepromatous (multibacillary) leprosy. *Int. J. Dermatol.* **44(7),** 576–578.
2. Khan, T., Awan, A.A., Kazmi, H.S., Shah, A.A., Muhammad, S., and Muhammad, S. (2002) Frequency of ocular complications of leprosy in institutionalized patients in NWFP Pakistan. *J. Ayub Med. Coll. Abbottabad* **14(4),** 29–33.
3. Sehgal, V.N. (1994) Leprosy. *Dermatol. Clin*. **12(4),** 629–644.
4. Thompson, A.M., Lynn, A.A., Robson, K., Joyce, M.P., Fivenson, D.P., and Scollard, D. (2003) Lepromatous phlebitis of the external jugular vein. *J. Am. Acad. Dermatol.* **49(6),** 1180–1182.

**This article should be cited as follows:**

Yaghoobi, R., Feily, A., Ranjbari, N., Lotfi, J., and Yaghoobi, E. (2010) Lepromatous leprosy: a commonly misdiagnosed disease. *TheScientificWorldJOURNAL* **10**, 2348–2349. DOI 10.1100/tsw.2010.206.
